# Supplementary material for: Common irrigation drivers of freshwater salinisation in river basins worldwide
Source: Nat Commun. 2021 Jul 9;12:4232. doi: 10.1038/s41467-021-24281-8 (PMC8270903; doi:10.1038/s41467-021-24281-8)
Supplement: Supplementary file 1 — Supplementary Information [file 41467_2021_24281_MOESM1_ESM.pdf]

Supplementary information for

Common irrigation drivers of freshwater salinisation in river basins worldwide

Josefin Thorslund[1,2*], Marc F.P. Bierkens [2,3], Gualbert H.P. Oude Essink[2,3], Edwin H. Sutanudjaja[2], Michelle T.H. van Vliet[2]

[1] Department of Physical Geography and the Bolin Centre for Climate Research, Stockholm University, Sweden

[2] Department of Physical Geography, Utrecht University, The Netherlands

[3] Unit Subsurface and Groundwater Systems, Deltares, The Netherlands

[*]Correspondence to: josefin.thorslund@natgeo.su.se

The supplementary information is presented in the following sections:

(S1) Salinity monitoring data availability

(S2) Driver variables processing

(S3) Salinity patterns and significance to driver variables

(S4) Random Forest variable selection and performance assessment

S1. **Salinity monitoring data availability**

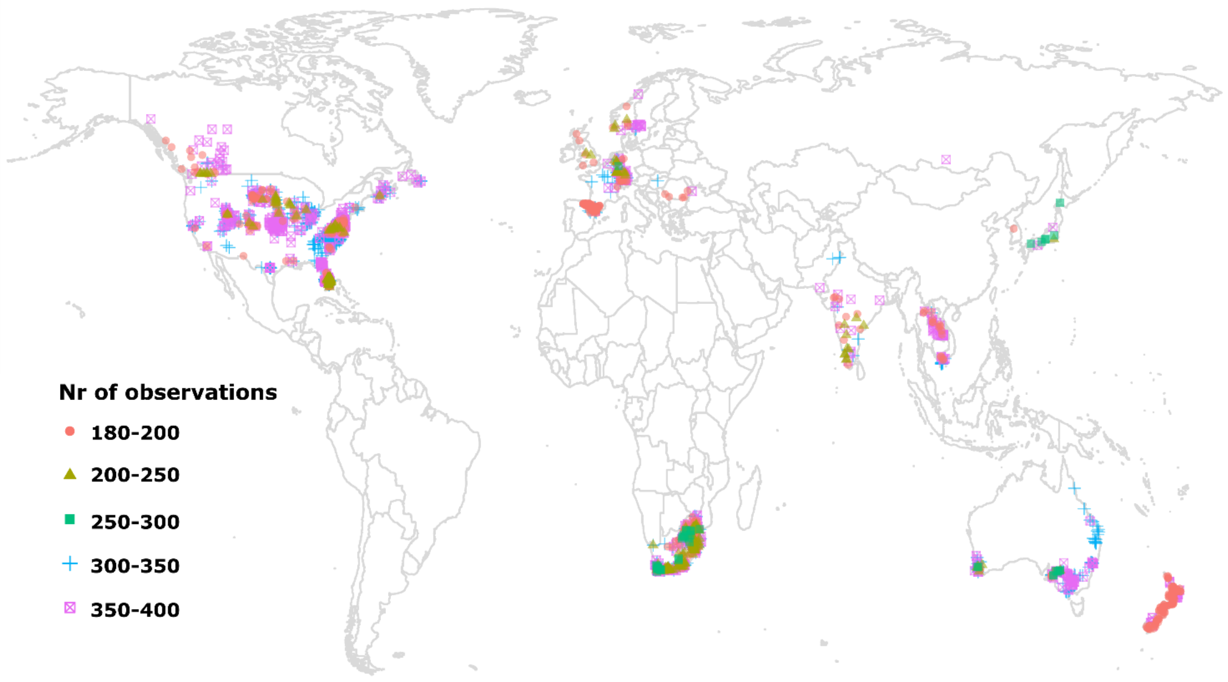

Supplementary Figure 1. Global monthly aggregated salinity observations. Map showing the number of monthly salinity observations at each river monitoring location of the global salinity dataset[1], combined with data from GEMStat[2] and Mekong River commission data portal[3], over the 1980-2010 period. The lowest category represents the selection criteria of a minimum of 15 years (180 months) of data availability.

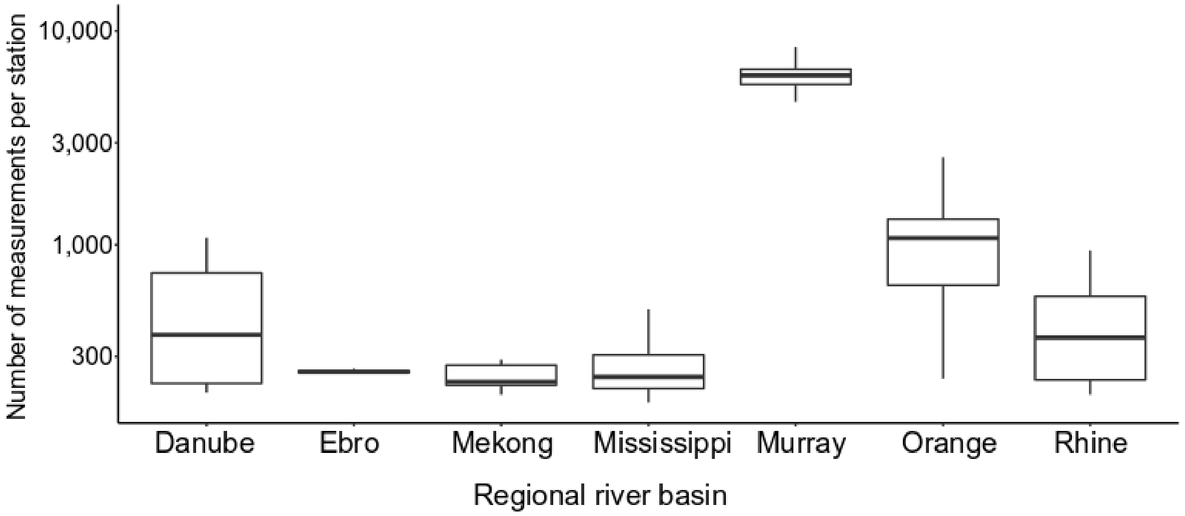

Supplementary Figure 2. Distribution of total salinity measurements within the studied regional river basins. Boxplots showing the distribution of salinity measurements available per station (sub-basin) within the selected seven regional river basins. Boxplot statistics include the median (thick vertical black lines), interquartile range (IQR: 25th percentile; Q1 and 75th percentile; Q3) and whiskers (confidence interval of Q1 - 1.5∗IQR to Q3 + 1.5∗IQR).

S2. **Driver variables processing**

Human drivers (agricultural-related)

Irrigation and non-irrigation water withdrawals and return flows

Sector specific water withdrawals and return flows over each sub-basin were computed using monthly outputs from the PCR-GLOBWB 2 model[4], over the 1980-2010 period and at 5x5 arc-minute spatial resolution. All water-use simulations follow the ISIMIP2a protocol; from the Inter-Sectoral Impact Model Intercomparison Project[5] and are based on historical socio-economic scenarios (hist_varsoc), which have been computed by taking climate, population, GDP, land-use, and technological developments into account[5]. The water allocation data simulated by PCR-GLOBWB 2 has been validated to both reported values and to independent assessments on country, continent and global scales[4,6]. Specifically, we used monthly outputs of (i) actual irrigation water withdrawal (Irr. ww), as well as actual water withdrawals from domestic, industrial and livestock sectors, from which we computed (ii) total non-irrigation water withdrawals (Non-irr. ww). We also gathered monthly timeseries of actual water consumption data, for the same sectors and used this data to calculate irrigation and non-irrigation return flows (Irr. Rf, Non-irr. rf), through subtracting model output data of withdrawal from consumption over each grid cell, and then summarizing all grid cells within each sub-basin polygon over each time-period (month).

Cropland area (total- and irrigated)

Two different data products were used to estimate annual total cropland (Tot. cropland), as well as the ratio of irrigated area to total sub-basin area (Norm. irr. area), over the 1980-2010 period. The MIRCA 2000 dataset[7] was used to get an estimate the ratio of irrigated cropland to total cropland area. This globally gridded dataset contains information on irrigated and rainfed crop areas, with 26 crop classes for each month around the year 2000. The ratio was estimated using an average of all 26 crop classes and associated grid areas, for both irrigated versus rainfed crops. Although reported uncertainties relating to crop seasonality do exist, information on the sum of harvested area for irrigated and rainfed crops, as well as the sum of irrigated area for all crops have shown to correlate well to other datasets[7]. Monthly data was summarized into an annual average, giving us a globally gridded ratio of irrigated cropland tot total cropland, at a 5 by 5 arc-minute grid cell resolution for the year 2000. We used this ratio (assuming the ratio in the year 2000 to be representative for all years) to estimate annual average total cropland, as well as total irrigated cropland over each sub-basin, over each year within the 1980-2010 period. To do this, we combined the first ratio estimate from MIRCA, with the NOAA-HYDE dataset, which contains annual gridded cropland area, also at a global gridded spatial resolution of 5 by 5 arc-minute[8,9]. This dataset was developed using widely accepted global reconstruction of cropland and pastureland, in combination with common wood harvest and urban land data sets. The NOAA-HYDE dataset contains estimates of the percentage of each grid cell area containing 28 different land-cover types, including the only two cropland-specific classes (named C3crop and C4crop). These cropland classes were added together, over each year of our study period. From this, we then extracted timeseries of annual total cropland over each sub-basin, by multiplying the cropland percentages from the NOAA-HYDE dataset with each sub-basin area. This total cropland area was then used to estimate total irrigated area, by multiplying it with the year 2000 irrigated crop ratio, developed from the MIRCA 2000 dataset as described above.

Fertilizer use

Fertilizer use (Nitrogen and Phosphorous) over each sub-basin was calculated, using the gridded nitrogen and phosphorus fertilizer use for global agriculture production data set[10,11]. This global dataset contains time-series gridded data of annual N and P fertilizer use rate (g m^{-2}), at a 0.5°x 0.5° resolution for the period of 1900-2013. This gridded dataset has been compared and matched with historical land use maps from HYDE 3.2[12], as well as country level crop-specific N and P fertilizer use rates from the IFA[13]. We extracted data for the years 1980-2010 and estimated average fertilizer use over each sub-basin, and from this we calculated basin total fertilizer use for (i) Nitrogen (N. total) (ii) Phosphorous (P. total) and (iii) Total (sum of (i) and (ii); Tot. Fert.).

Dams/reservoirs

Reservoirs are an important water supply source for irrigation[14] and could contribute to increasing freshwater salinity levels through evapo-concentrations effects caused by increased relative evapotranspiration from the open water surfaces of reservoirs[15]. We quantify the presence and size of dams and associated reservoirs in each sub-basin, by using the Global Reservoir and Dam Database (GRanD) v.1.1[16]. This dataset includes 6,862 records of reservoirs and dams, from which 1,276 were included within our study regions. The GRanD dataset has been validated through extensive cross-validation, error checking, and identification of duplicate records and missing information has been completed, using a multitude of sources and statistical approaches[16]. We estimated for each sub-basin (i) total dam capacity (Dam storage), (ii) number (n_dams) (iii) ratio of total reservoir capacity to basin area (Norm. dam storage) and (iv) ratio of total dam area to basin area (Norm. dam area). Pre-processing was first done in ArcMap, by pairing all dams to associated sub-basin, using "spatial join", with the sub-basins layer as target feature and the dam layer as the join feature, and the "join one to many" option, using the "contains" join operation. After this join was done, the data was exported to R, where further sub-basin calculations were done.

Hydro-climatic drivers

Temperature, precipitation and evapotranspiration

We used the CRU TS (Version 4) high-resolution gridded climate dataset[17] to calculate monthly timeseries, as well as long-term annual average temperature (T) over each derived sub-basin. This dataset covers a global 0.5°x 0.5° grid and is based on interpolation of monthly climate anomalies from widespread networks of weather station observations. Temperature data was extracted from monthly average values, over the 1980 – 2010 period and spatially averaged over each sub-basin, using the R function "exact_extract" of the raster package.

Additionally, monthly timeseries of Precipitation (P), actual- and potential evapotranspiration (AET and PET), as well as Evaporative ratios; (PET/P and AET/P) were extracted and computed over each sub-basin, using model output data from the global hydrological model PCR-GLOBWB 2[4]. These model runs were produced using historical simulations following the ISIMIP2a protocol[5]. The ISIMIP framework includes bias-corrected global climate model output data sets on a 0.5°x 0.5° global grid. This climate data is based on the reanalysis data sets ERA-40 (for WATCH) and ERA-Interim (for WFDEI). The data set

covers the period 1901-2012, where the data for 1901-1978 are taken from WATCH, and from 1979 onwards from WFDEI[18,19].

Discharge

Monthly as well as long-term annual average discharge (*Q*) over each sub-basin were quantified, using model outputs of the PCR-GLOBWB 2[4], with runs made under the ISIMIP2a protocol[5] for WATCH forcing data Era Interim (WFDEI)[18]. Monthly gridded (5x5 arc-minute resolution) discharge over the 1980-2010 period was produced through accounting for the historical evolution of irrigated areas, dams and reservoirs, to obtain a more realistic estimate of the historical evolution of runoff and discharge. The PCR-GLOBWB 2 model has been shown to realistically predict river discharge, through comparisons with in-situ discharge observations from the Global Runoff Data Centre[4]. We aggregated the values of each grid-cell within each sub-basin polygon, over each month, to get total sub-basin specific monthly discharge timeseries over the considered time period of 1980-2010.

Geographic drivers

Soil salinity

Data on soil salinity was extracted for each sub-basin, using the WISE30sec dataset[20]. The WISE contains soil properties for the world, at a 30 by 30 arc-second resolution and was developed from soil profile observational data[21] in combination with an overlay of the Harmonized World Soil Database[22] and the Köppen–Geiger climate zones map as categorical co-variate. The WISE30sec dataset contains soil information at different depths, up to a depth of 2 m, for 20 different soil properties. For this study, we used electrical conductivity (EC) information, for the top two soil layers; (i) layer D1 (0-20 cm from the surface; EC top soil) and (ii) layer D2 (20-40 cm from the surface; EC sub soil). EC information was extracted for these layers from the main Microsoft access database and joined to the raster data, using the "join" data attribute tables (the join was based on the NEWSUID column). The "lookup" function of the spatial analyst package was used to create a new raster for each soil layer, based on the value in the attribute table corresponding to the EC values (dS m^{-1}). These two new rasters were then further processed in R and EC values were extracted for each of the topsoil and subsoil layers, and a weighted average of the two layers was used to determine (iii) the mean soil salinity at each pixel (EC soil aver.). Sub-basin scale soil salinity was then obtained as the spatially averaged values over each sub-basin.

Elevation

The absolute elevation (Elevation) of each EC river measurement location (i.e. the outlet of each sub-basin) was extracted in ArcMap, using the 15 Arc-Seconds Hydrologically Conditioned DEM raster dataset, from Hydrosheds[23], to allow for consistency with the basin delineation procedure. We extracted the values using the "extract values to points" of the spatial analyst toolbox.

Distance from coast (absolute and relative)

The absolute distance to the coast (Dist. coast) from each river salinity monitoring location was estimated in ArcMap, using the vector map from Natural Earth (https://www.naturalearthdata.com/downloads/110m-physical-vectors/110m-coastline/), a 1:110 m scale resolution of the coastline. We used the "near table" function to pair the EC stations to the vector

layer. In addition to this variable, we also calculated a composite variable of relative distance from coast (Rel. dist. coast), which was estimated according to equation (1);

$$\mathrm{Rel}_{\mathrm{dist\ coast}} = \frac{1}{\mathrm{distance\ to\ coast}} \cdot \frac{1}{\mathrm{absolute\ elevation}} \qquad (1)$$

This variable represents a relative elevation compared to sea level and was created with the purpose of highlighting potential tidal effects, and/or stronger surface- and groundwater interactions. The underlying assumption of this variable is of higher groundwater-surface water interactions (i.e. seepage)[24] for lower elevation locations close to the ocean, compared to locations at higher elevation, further upstream locations.

S3. Salinity patterns and significance to driver variables

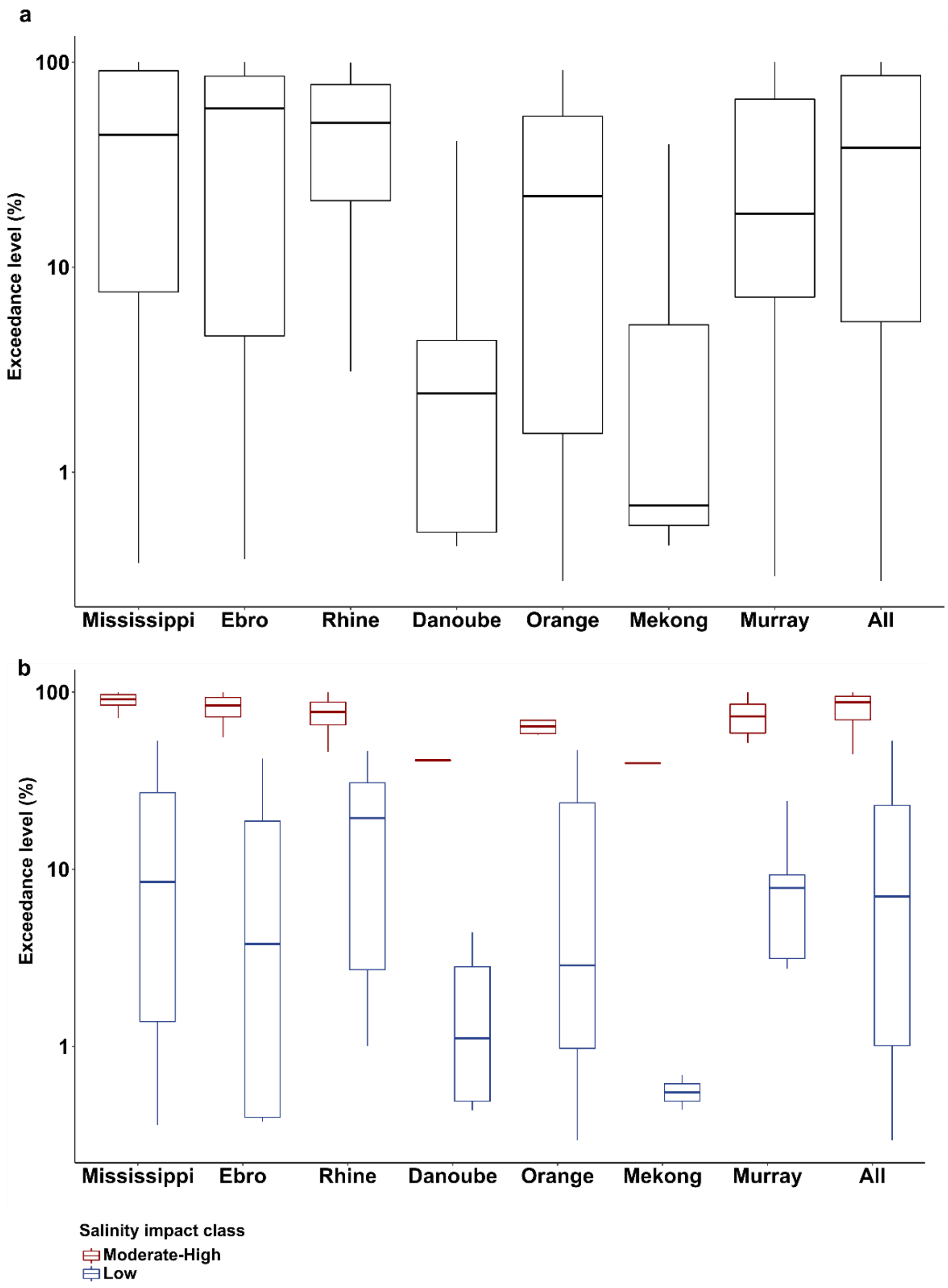

Supplementary Figure 3. Distribution of sub-basin salinity threshold exceedance level within each regional basin. Boxplot showing exceedance level (% of months compared to total that each sub-basin exceeds the salinity threshold) within each regional river basin, as well as for all sub-basins together (panel a), and (panel b) grouped over sub-basins within the Low salinity impact class (i.e. sub-basins with long term annual average salinity values below 700 µS cm^{-1}), and sub-basins within the Moderate-High salinity impact class (i.e. long-term salinity levels above this threshold). Boxplot statistics include the median (thick vertical lines), interquartile range (IQR: 25th percentile; Q1 and 75th percentile; Q3) and whiskers (confidence interval of Q1 - 1.5∗IQR to Q3 + 1.5∗IQR).

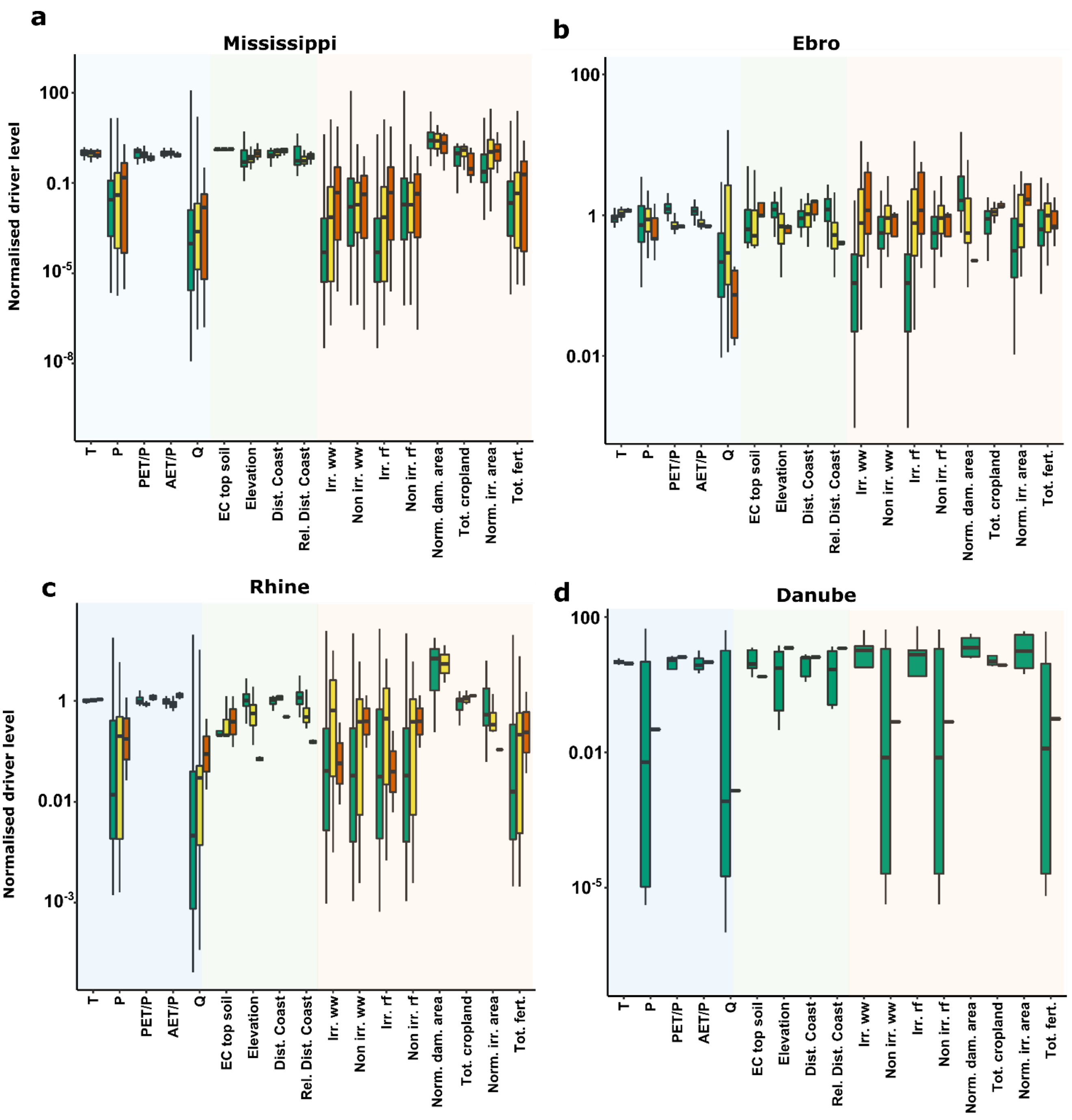

a
Mississippi
b
Ebro
c
Rhine
d
Danube
Normalised driver level
100
0.1
10-5
10-8
1
0.01
10-3
10-5
T
P
PET/P
AET/P
Q
EC top soil
Elevation
Dist. Coast
Rel. Dist. Coast
Irr. ww
Non irr. ww
Irr. rf
Non irr. rf
Norm. dam. area
Tot. cropland
Norm. irr. area
Tot. fert.

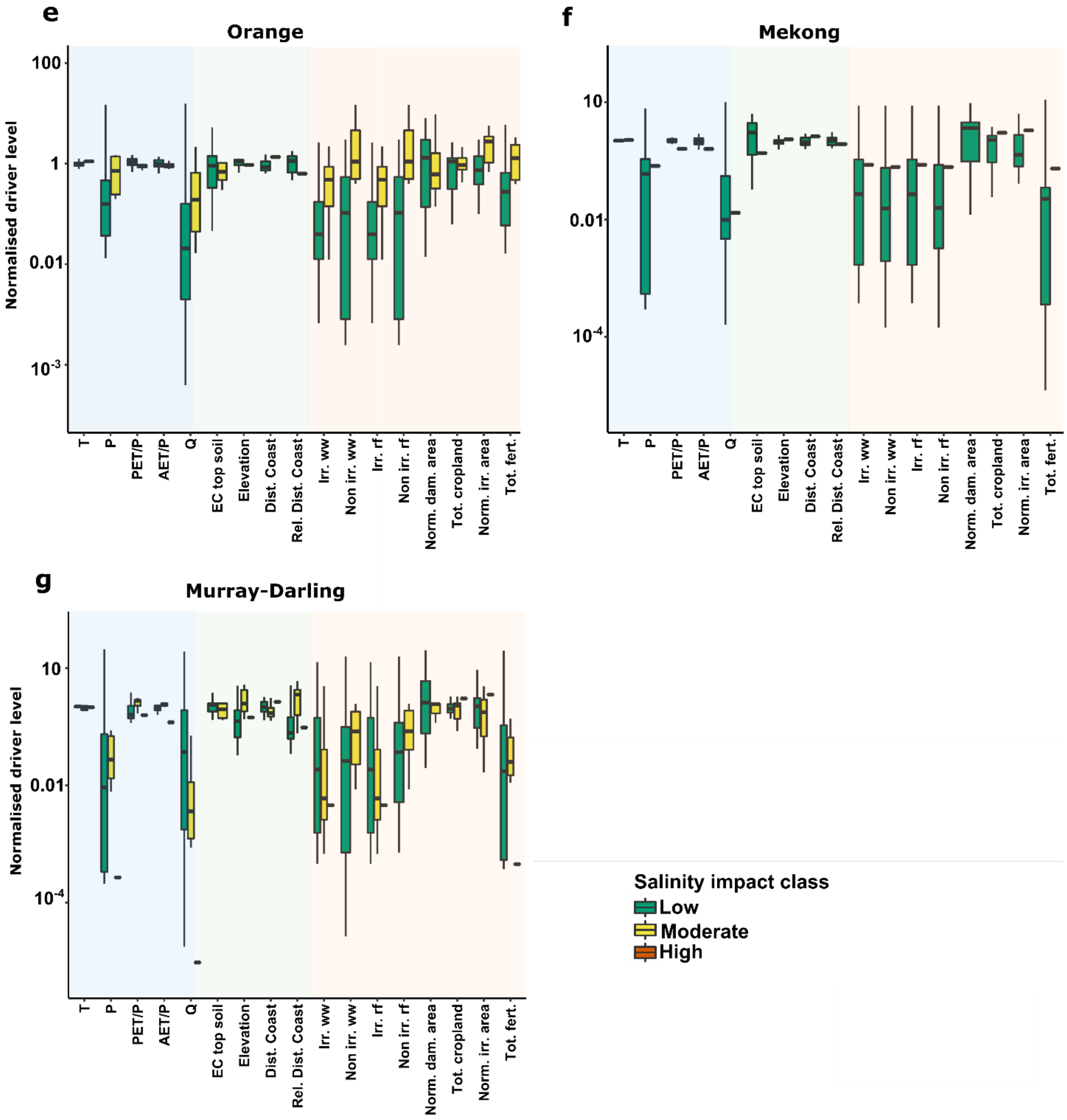

Supplementary Figure 4. Regional contributions of hydroclimatic, geographic and human drivers across sub-basin salinity impact classes. The figures show a selection of 17 out of the total 26 considered driver variables, over all sub-basins within each regional river basin. The selection includes all driver categories (as listed in Table 1), but where multiple variables within the same category exists (e.g. for dams), only the variable showing statistically significant results was included (all summary statistics are included in Supplementary Table 1).The salinity impact classes are based on groups of sub-basins with Low (EC < 700 µS cm^{-1}; green boxplots), Moderate (EC = 700 – 1500 µS cm^{-1}; orange boxplots) and High (EC > 1500 µS cm^{-1}; red boxplots) salinity levels, classified from long-term annual average values. The selected drivers are plotted along groups of (i) hydroclimatic, (ii) geographic and (iii) human drivers on the x-axis, and their normalized long-term annual average levels on the y-axis. Note that each driver level is to be compared across the salinity impact classes (green-yellow-orange boxplots). Boxplot statistics include the median (vertical thick black lines), interquartile range (IQR: 25th percentile; Q1 and 75th percentile; Q3) and whiskers (confidence interval of Q1 - 1.5∗IQR to Q3 + 1.5∗IQR).

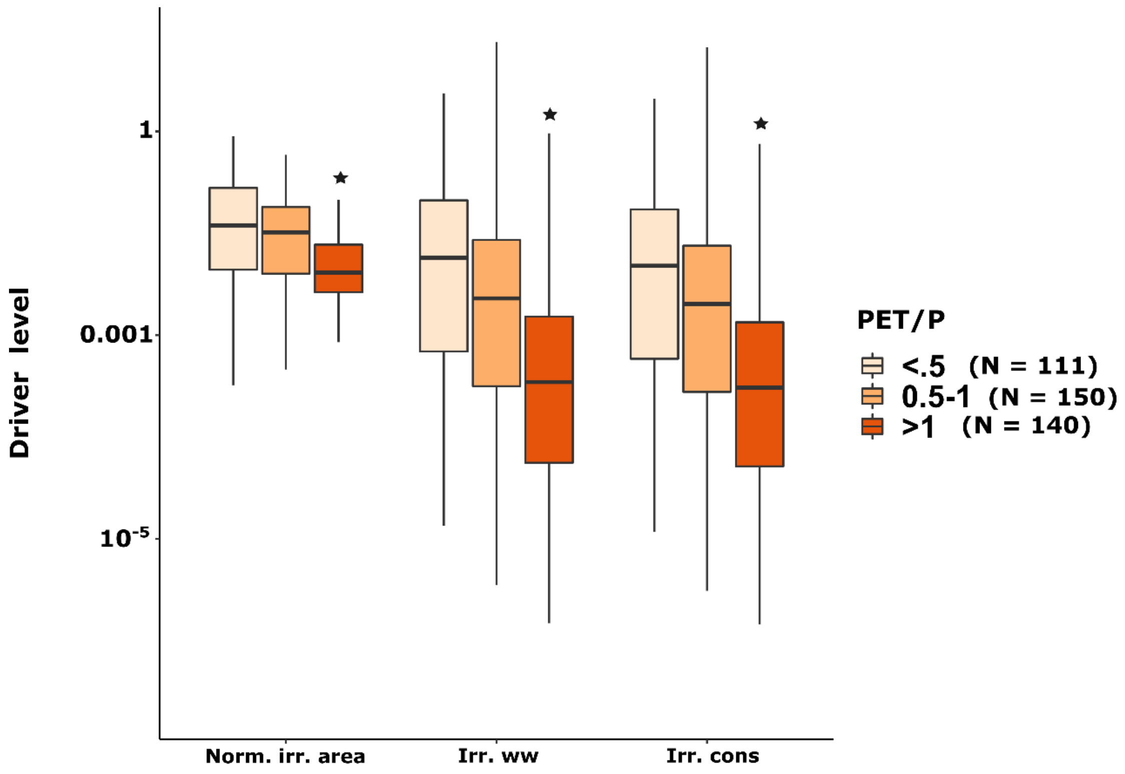

Supplementary Figure 5. Relationships between sub-basin irrigation specific variables and aridity. Boxplots showing the distribution of sub-basin irrigated area (Norm. Irr. area) irrigation water use (irrigation water withdrawals; Irr. ww and irrigation consumption; Irr. cons), grouped over sub-basin aridity index (PET/P). The sub-basin aridity index classes are divided into three groups of similar sub-basin group sizes (N) and range from lower (<0.5) to higher (0.5-1 and >1) aridity, classified from long-term annual average values over the 1980-2010 period. Boxplot statistics include the median (vertical thick black lines), interquartile range (IQR: 25th percentile; Q1 and 75th percentile; Q3) and whiskers (confidence interval of Q1 - 1.5∗IQR to Q3 + 1.5∗IQR). The stars (*) over the boxplots indicate where there are statistically significant differences in the irrigation related driver levels between low-high aridity groups (for <0.5 and > 1 groups; statistics based on Wilcoxon rank sum tests with a significance difference identified the 95% significance level, p<0.05).

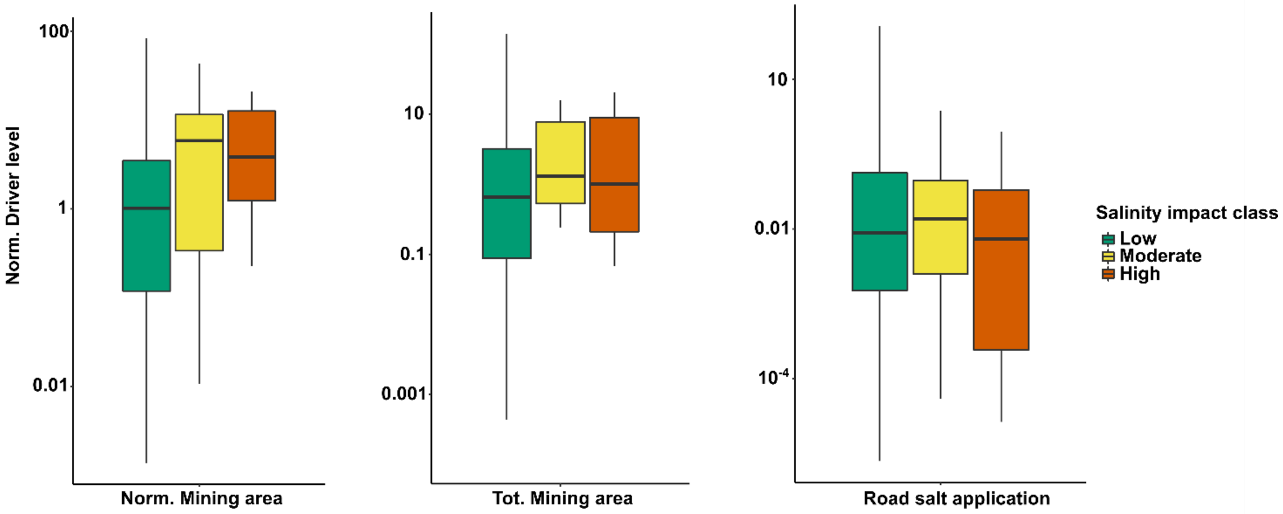

Supplementary Figure 6. Contributions of mining and road salt drivers across sub-basin salinity impact levels. Boxplots showing the distribution of sub-basin mining area (Norm. Mining area and Tot. Mining area)[25] and road salt application (in pounds; annual average application over the years 1992-2010)[26] grouped over sub-basin salinity impact classes. The mining area drivers are quantified for all sub-basins (N=401), whereas the road salt driver is quantified over the Mississippi sub-basins as a case study example (N=167). Boxplot statistics include the median (thick vertical black lines), interquartile range (IQR: 25th percentile; Q1 and 75th percentile; Q3) and whiskers (confidence interval of Q1 - 1.5∗IQR to Q3 + 1.5∗IQR).

Supplementary Table 1. Significance between driver mean levels and salinity. Results of the Wilcoxon rank sum test for mean levels of each driver variable between salinity impact classes. The table shows the p-values for the Wilcoxon test applied to each driver, over all possible salinity impact class combinations. Statistically significant differences (p<0.05) are shown in bold.

Driver/ Salinity impact class	Low-High	Low-Moderate	Moderate-High
T	**0.007***	0.657	**0.031**
P	0.429	0.353	0.615
PET	0.218	0.176	0.396
AET	0.236	0.207	0.385
PET/P	**0.003**	**0.024**	0.182
AET/P	**0.0003**	0.092	**0.002**
Q	0.806	0.203	0.643
Irr. ww	**0.004**	**0.0003**	0.330
Non-irr. ww	0.328	**0.037**	0.099
Irr. rf	**0.005**	**0.0005**	0.320
Non-irr. rf	0.373	**0.036**	0.107
Dam. storage	0.772	0.678	0.560
Nr. dams	0.587	0.490	0.373
Norm. dam storage	0.991	0.751	0.849
Norm. dam area	0.965	0.872	0.903
Tot. cropland	0.223	**0.0008**	**0.003**
Norm. irr. area	0.006	**0.00002**	0.842
N. total	0.064	**0.015**	0.380
P. total	0.158	**0.013**	0.721
Tot. fert.	0.076	**0.015**	0.434
EC top soil	0.258	**0.033**	0.733
EC sub soil	0.517	0.251	0.157
EC soil aver.	0.897	0.178	0.277
Elevation	0.200	0.224	**0.024**
Dist. coast	**0.0001**	0.746	**0.0003**
Rel. dist. coast	0.063	0.771	0.109

*For interpretation of the direction of the significant differences between each driver and the salinity impact class, the reader is referred to Fig. 4. For description of the driver variable definitions, the reader is referred to Table 1.

Supplementary Table 2. Results of the Wilcoxon rank sum test for mean levels of each driver variable between two new salinity impact classes, representing groups of sub-basins below and above the threshold of 700 µS cm^{-1}. The table shows p-values for the Wilcoxon rank sum test applied to the two impact classes. Significant differences ($p<0.05$) are shown in bold.

Driver/ Salinity impact class	Below-Above*
T	0.140
P	0.268
PET	0.100
AET	0.121
PET/P	**0.001**
AET/P	**0.004**
Q	0.239
Irr. ww	**0.00002**
Non-irr. ww	0.170
Irr. rf	**0.00004**
Non-irr. rf	0.170
Dam. storage	0.816
Nr. dams	0.724
Norm. dam storage	0.785
Norm. dam area	0.906
Tot. cropland	**0.020**
Norm. irr. area	**0.000002**
N. total	**0.005**
P. total	**0.008**
Tot. fert.	**0.006**
EC top soil	**0.023**
EC sub soil	0.478
EC soil aver.	0.277
Elevation	0.608
Dist. coast	0.059
Rel. dist. coast	0.318

* The salinity classes are here divided into two new impact classes; "below" (N=262) and "above" (N=139), based on threshold levels of 700 µS cm^{-1} (representing the lowest threshold level for irrigation water use).

S4. Random Forest variable selection and performance assessment

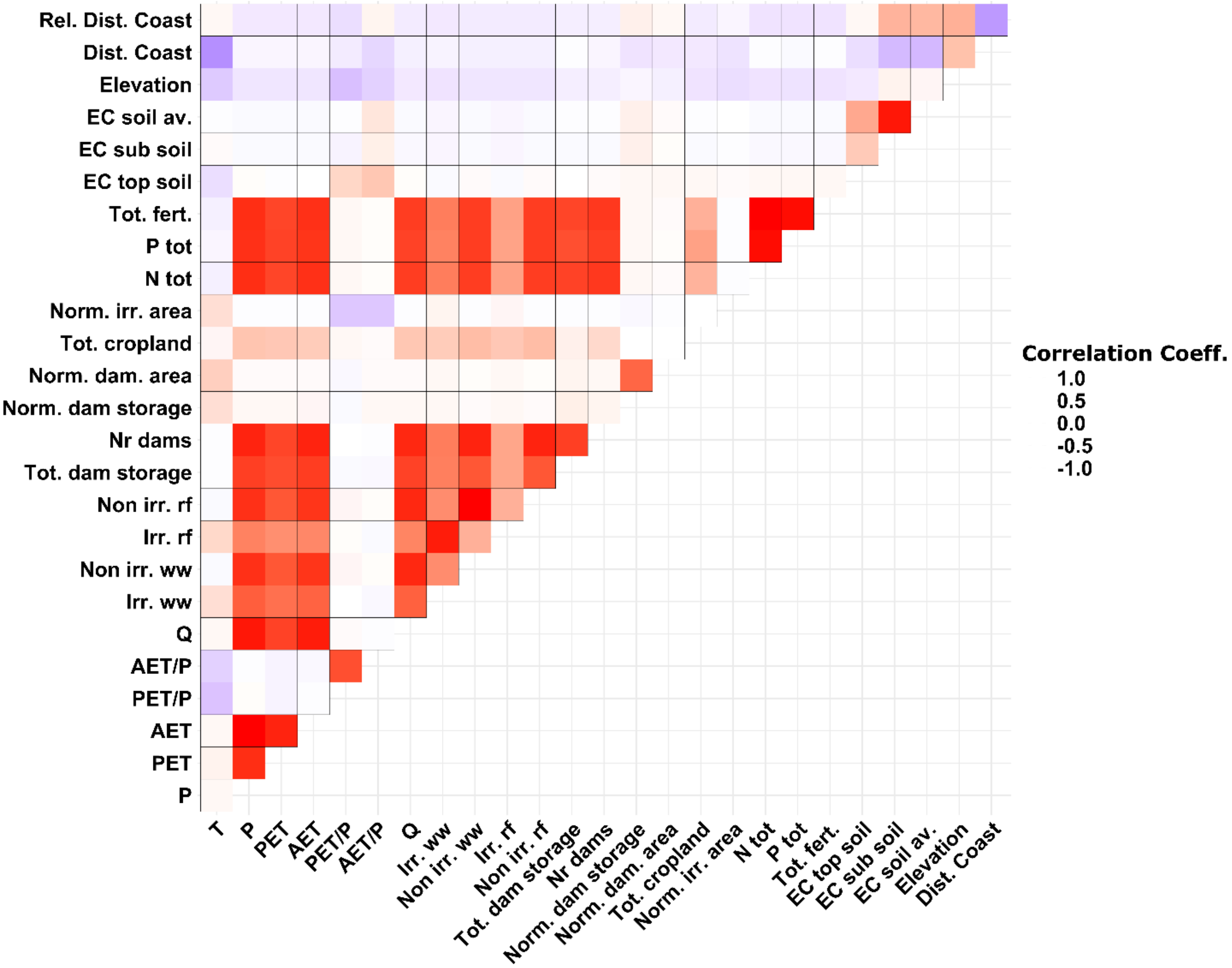

Supplementary Figure 7. Matrix display of the Pearson correlation between all driver variables, based on long-term annual average values. Each cell of the matrix shows the correlation between two driver variables; positive correlations are displayed in red-orange colours and negative correlations in blue-purple colours.

Supplementary Table 3. Random Forest model final driver variable selection. For explanation of driver variable abbreviations, the reader is referred to Table 1. The variables with "trend" included after the abbreviated name represent the trend magnitude (Sen's slope value) of that variable.

Random forest model	Driver variables
1) All sub-basins with increasing salinity trends (N=128)	Norm. irr. area; Dist. coast; EC top soil; Rel. dist. coast; Elevation; EC soil av.; Q trend; EC sub soil; Irr. ww; T trend; PET/P; AET/P; Irr. rf.; T; Irr. rf trend
2) All sub-basins with decreasing salinity trends (N=96)	Dist. coast; Non-irr. rf; Non-irr. ww; AET/P trend; PET/P trend; Q trend

Supplementary Table 4. Random Forest model accuracy. Accuracy parameters include the squared correlation coefficient (R^2), the Root Mean Squared Error (RMSE), the Mean Absolute Error (MAE) and normalized MAE (NMAE).

Random forest model	R^2	RMSE ($\mu S\ cm^{-1}$)	MAE ($\mu S\ cm^{-1}$)	NMAE
1) All sub-basins with increasing salinity trends (N=128)	0.61	224	165	0.27
2) All sub-basins with decreasing salinity trends (N=96)	0.51	466	351	0.46

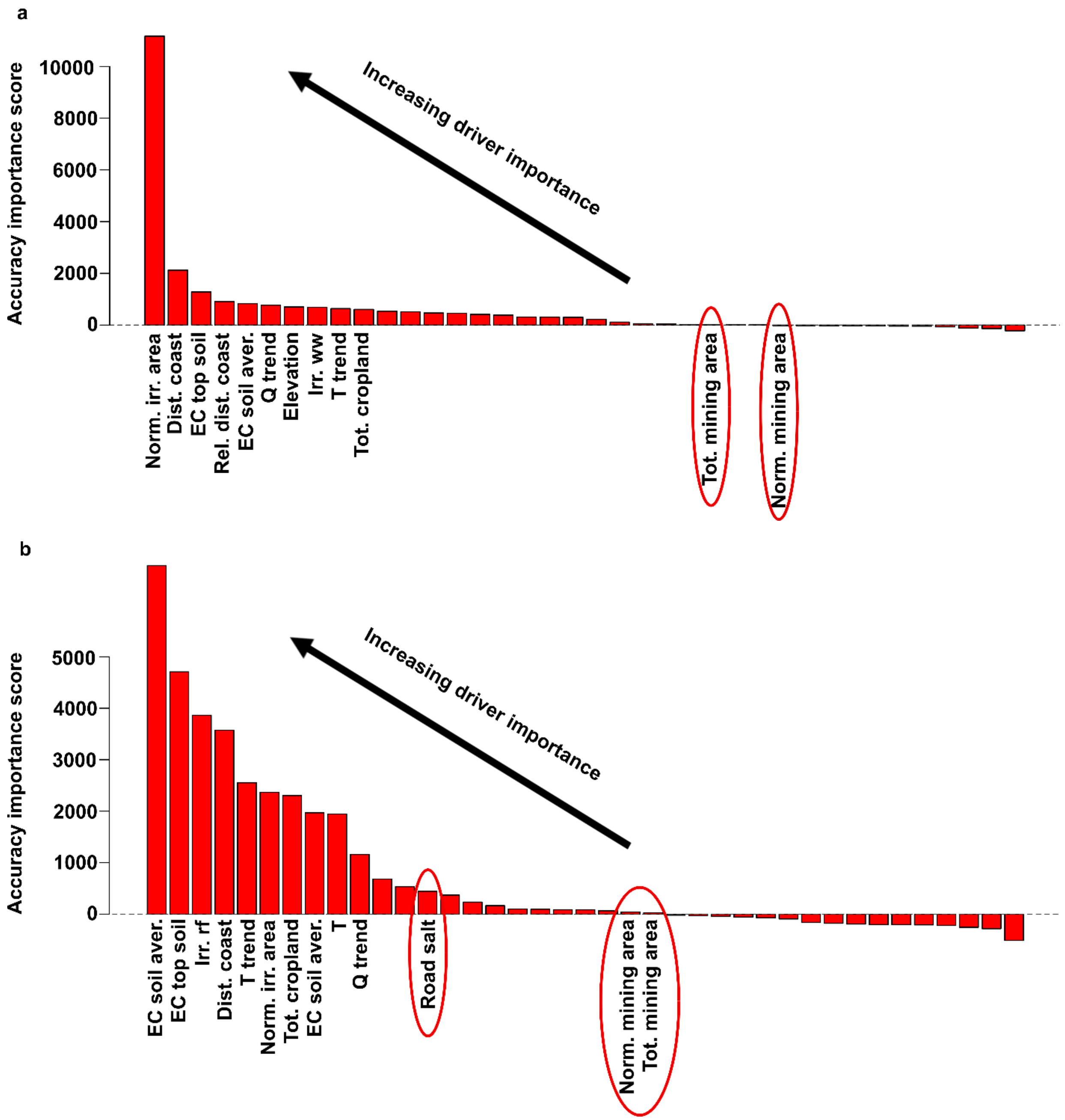

Supplementary Figure 8. Relative driver contributions, including mining and road salt drivers, for predicting salinity levels in sub-basins with increasing salinisation. Results from the CPI analysis when including mining (sub-basin normalized mining area and total mining area) and road (sub-basin total annual application) salt as predictor variables in the RF model to predict salinity levels in sub-basins experiencing salinisation. Bar charts show the relative importance of driver variables in (a) all sub-basins and (b) for sub-basins within the Mississippi river basin (a relevant case study for road salt application), using the conditional permutation importance method within the Random Forest approach. For illustration purposes, only the top 10 drivers, as well as the relative importance of mining and road salt are shown. The accuracy importance score (y-axis; unitless) represent the relative importance of each predictor variable in achieving the prediction capacity of the Random Forest model (note the different y-axes in panel a and b and that the absolute values of the scores should not be interpreted, only the relative scores between drivers).

Supplementary References

1. Thorslund, J. & van Vliet, M. T. H. A global dataset of surface water and groundwater salinity measurements from 1980–2019. *Scientific Data* **7**, 231 (2020).
2. GEMStat data portal (United Nations Environment Programme, 2018) GEMS/Water. http://www.gemstat.org/
3. Mekong River commission data portal, MRC. https://www.mrcmekong.org/
4. Sutanudjaja, E. H. et al. PCR-GLOBWB 2: a 5 arcmin global hydrological and water resources model. *Geosci. Model Dev.* **11**, 2429–2453 (2018).
5. Warszawski, L. et al. The Inter-Sectoral Impact Model Intercomparison Project (ISI–MIP): Project framework. *PNAS* **111**, 3228–3232 (2014).
6. de Graaf, I. E. M., van Beek, L. P. H., Wada, Y. & Bierkens, M. F. P. Dynamic attribution of global water demand to surface water and groundwater resources: Effects of abstractions and return flows on river discharges. *Advances in Water Resources* **64**, 21–33 (2014).
7. Portmann, F. T., Siebert, S. & Döll, P. MIRCA2000—Global monthly irrigated and rainfed crop areas around the year 2000: A new high-resolution data set for agricultural and hydrological modeling. *Global Biogeochemical Cycles* **24**, GB1011 (2010).
8. Jain, A. K., Meiyappan, P., Song, Y. & House, J. I. CO2 emissions from land-use change affected more by nitrogen cycle, than by the choice of land-cover data. *Global Change Biology* **19**, 2893–2906 (2013).
9. Meiyappan, P. & Jain, A. K. Three distinct global estimates of historical land-cover change and land-use conversions for over 200 years. *Front. Earth Sci.* **6**, 122–139 (2012).
10. Lu, C. & Tian, H. Global nitrogen and phosphorus fertilizer use for agriculture production in the past half century: shifted hot spots and nutrient imbalance. *Earth System Science Data* **9**, 181–192 (2017).
11. Lu, C. & Tian, H. Half-degree gridded nitrogen and phosphorus fertilizer use for global agriculture production during 1900-2013. Pangaea https://doi.pangaea.de/10.1594/PANGAEA.863323 (2014).
12. Klein Goldewijk, K., Beusen, A., Doelman, J. & Stehfest, E. Anthropogenic land use estimates for the Holocene – HYDE 3.2. *Earth System Science Data* **9**, 927–953 (2017).
13. Heffer, P. Gruère, A & Roberts, T. Assessment of fertilizer use by crop at the global level 2010–2010/11 (International Fertilizer Industry Association, Paris, 2013).

14. Yoshikawa, S., Cho, J., Yamada, H. G., Hanasaki, N. & Kanae, S. An assessment of global net irrigation water requirements from various water supply sources to sustain irrigation: rivers and reservoirs (1960–2050). *Hydrol. Earth Syst. Sci*. **18**, 4289–4310 (2014).
15. Jaramillo, F. & Destouni, G. Local flow regulation and irrigation raise global human water consumption and footprint. *Science* **350**, 1248–1251 (2015).
16. Lehner, B., C. et al. Global Reservoir and Dam Database, Version 1 (GRanDv1): Dams, Revision 01. Palisades, NY: NASA Socioeconomic Data and Applications Center (SEDAC). https://doi.org/10.7927/H4N877QK. Accessed 5th Aug 2020. (2011).
17. Harris, I., Osborn, T. J., Jones, P. & Lister, D. Version 4 of the CRU TS monthly high-resolution gridded multivariate climate dataset. *Scientific Data* **7**, 109 (2020).
18. Weedon, G. P. et al. The WFDEI meteorological forcing data set: WATCH Forcing Data methodology applied to ERA-Interim reanalysis data. *Water Resources Research* **50**, 7505–7514 (2014).
19. Weedon, G.P. et al. Creation of the WATCH Forcing Data and Its Use to Assess Global and Regional Reference Crop Evaporation over Land during the Twentieth Century. J. *Hydrometeor.,* **12**, 823–848 (2011).
20. Batjes N. H. Harmonized soil property values for broad scale modelling (WISE30sec) with estimates of global soil carbon stocks. *Geoderma* **269**, 61-68 (2016).
21. Batjes, N. H. Harmonized soil profile data for applications at global and continental scales: updates to the WISE database. *Soil Use and Management* **25**, 124–127 (2009).
22. Fischer, G., F. et al. Global Agro-Ecological Zones Assessment for Agriculture (IIASA, Laxenburg, Austria and FAO, Rome, Italy 2008).
23. Lehner, B., Verdin, K. & Jarvis, A. New global hydrography derived from spaceborne elevation data. *Eos, Transactions, American Geophysical Union* **89**, 93-94 (2008).
24. De Louw, P.G.B. Saline seepage in deltaic areas: Preferential groundwater discharge through boils and interactions between thin rainwater lenses and upward saline seepage (Vrije Universiteit Amsterdam, 198 p. 2013).
25. Maus, V. et al. A global-scale data set of mining areas. *Scientific Data* **7**, 289 (2020).
26. Bock, A. R. Falcone, J. A., & Oelsner, G. P. Estimates of Road Salt Application across the conterminous United States (1992-2015). 1992-2015: U.S. Geological Survey data release, https://doi.org/10.5066/P96IX385. (2018).
